# Complete mitochondrial genomes of two species of *Stichopathes* Brook, 1889 (Hexacorallia: Antipatharia: Antipathidae) from Rapa Nui (Easter Island)

**DOI:** 10.1080/23802359.2021.1990150

**Published:** 2021-10-15

**Authors:** Cynthia M. Asorey, Javier Sellanes, Daniel Wagner, Erin E. Easton

**Affiliations:** a Departamento de Biología Marina, Facultad de Ciencias del Mar and Sala de Colecciones Biológicas, Universidad Católica del Norte, Millennium Nucleus for Ecology and Sustainable Management of Oceanic Islands (ESMOI), Coquimbo, Chile; b Conservation International, Center for Oceans, Arlington, VA, USA; c School of Earth, Environmental, and Marine Sciences, University of Texas Rio Grande Valley, South Padre Island, TX, USA

**Keywords:** Black coral, Easter Island Ecoregion, Chile, mesophotic coral ecosystem, *Cirrhipathes*, *Antipathes*

## Abstract

We report the complete mitochondrial genomes of two antipatharian species, *Stichopathes* sp. SCBUCN-8849 and *Stichopathes* sp. SCBUCN-8850, collected between 120 and 180 m depth off Rapa Nui (∼ −27.1°, −109.4°). The size of the two mitogenomes are 20,389 bp (29.0% A, 15.2% C, 19.9% G, and 35.9% T) and 20,463 bp (29.0% A, 15.3% C, 19.9% G, and 35.8% T), respectively. Both mitogenomes have the classic Hexacorallia gene content of 13 protein-coding, two rRNA, and two tRNA genes plus a *COX1* intron with embedded *HEG* as found in the Antipathidae and other antipatharian families.

The order Antipatharia includes over 279 described species in seven families that occur in all oceans between 2 and 8600 m (Molodtsova and Opresko 2017). Antipathidae is composed of eight genera distinguished by morphological differences in colony branching pattern, polyps, and skeletal spines (Molodtsova and Opresko 2021). Recent phylogenetic studies based on the internal transcriber spacer 1 and 2 (*ITS1* and *ITS2*) rDNA (Bo et al. 2012) and mitochondrial genomes (Barrett et al. 2020) indicate that Antipathidae and several genera within Antipathidae are not monophyletic, highlighting the need for future taxonomic revisions.

During benthic surveys off Rapa Nui (Easter Island) two specimens of whip antipatharians with distinct colorations and morphologies (Supp. Mat.) were collected by chance, entangled in the propellers of a remotely operated vehicle. Specimen collection was performed under permissions Res. Ext N°41/2016 and N°3314/2017 from SUBPESCA (National Fishing Authority of Chile) to Universidad Católica del Norte (UCN) and from the “Consejo del Mar de Rapa Nui” (Rapa Nui Council of the Sea). A yellow morphotype was collected between 120 and 180 m (27.100° S; 109.431° W) and a red morphotype was collected at 180 m (27.101° S; 109.426° W). Specimens were stored in 95% ethanol and deposited in the Sala de Colecciones, UCN (SCBUCN, Javier Sellanes, sellanes@ucn.cl): SCBUCN-8849 (yellow) and SCBUCN-8850 (red). On both specimens, polyp arrangement was in a single row on only one side of the corallum, a diagnostic morphological character for the genus *Stichopathes* (Bo and Opresko 2015). Although morphological differences in skeletal spines characters, which are considered diagnostic for antipatharian species, were recorded (Supp. Mat.), neither could be assigned to described species by the authors or taxonomic experts consulted because of the poor quality or missing type material for many *Stichopathes* species, most of which were described over a century ago with limited information (Bo et al. 2012).

Isolated genomic DNA was submitted to Biopolymers Facility at Harvard Medical School for library preparation and next-generation sequencing (NextSeq 500). Trimmed reads (Trimmomatic-0.32, Bolger et al. 2014) were assembled *de novo* with SPAdes (Bankevich et al. 2012) on the University of New Hampshire Bioinformatics Core facility ron server. After circularizing and editing overlapping ends of the SPAdes contig in Geneious Prime 2021.1.1 (https://www.geneioius.com), trimmed reads (BBDuk v. 38.84) were mapped to the resulting reference sequence to generate a consensus sequence. Genes were annotated by manually adjusting *Stichopathes luetkeni* (NC018377) annotations mapped to the consensus sequence. Maximum-likelihood phylogenetic reconstructions were based on the concatenated mitochondrial protein-coding genes of these two *Stichopathes* specimens, 21 representatives of Antipatharia, and 8 representatives (outgroups) from Hexacorallia aligned with default MUSCLE (Edgar 2004) parameters. See Supp. Mat. for extended methods, including of *ITS1*-based reconstruction.

Both mitogenomes presented the classic pattern expected for Hexacorallia of 13 protein-coding genes, two rRNA genes, and two tRNA genes (tRNA^-met^ and tRNA^-trp^) plus a *COX1* intron with embedded *HEG* as found in Antipathidae and other antipatharian families (Barrett et al. 2020). The complete mitogenome of *Stichopathes* sp. SCBUCN-8849 (MZ157400) has 20,389 bp (29.0% A, 15.2% C, 19.9% G, and 35.9% T), whereas *Stichopathes* sp. SCBUCN-8850 (MZ157399) has 20,463 bp (29.0% A, 15.3% C, 19.9% G, and 35.8% T). These species were 98.71% similar (uncorrected p distances) across the entire mitogenome and 98.69% similar across the protein-coding genes. They form a well-defined clade with *S. luetkeni* and are sister to a clade containing the other three Antipathidae species and an Aphanipathidae species (Fig 1). Across the mitochondrial proteome, *Stichopathes* sp. SCBUCN-8849 and SCBUCN-8850 were respectively 99.25% and 98.25% similar to *S. luetkeni*. Based on ITS1 rDNA sequences, the Rapa Nui *Stichopathes* species were genetically distinct from each other (38.27% uncorrected p, MZ450123-MZ450124) based on 343-bp alignment of the *ITS1* region of 70 antipatharians and a scleractinian outgroup (Supp. Mat.). In the *ITS1*-based reconstruction, *Stichopathes* sp. SCBUCN-8849 groups within a clade mainly composed of species of the genus *Cirrhipathes* that differ from SCBUCN-8849 by 0.9-2.2%, whereas *Stichopathes* sp. SCBUCN-8850 groups within a clade (clade C in Bo et al. 2012) mainly composed of *Antipathes* species that differ from SCBUCN-8850 by 1.0–2.0%. These findings highlight the need for further phylogenetic studies and taxonomic revisions within Antipathidae. With only a few complete antipatharian mitogenomes available, the distinction of clades even at the family level is hindered (Barrett et al. 2020). Yet, phylogenetic information based on mitochondrial genomes is critical to understand evolutionary history and phylogeography and to provide insight into which taxa to prioritize for taxonomic revisions. A better understanding of their identity, evolutionary history, and distribution would, in turn, provide essential information for their conservation and management, especially in marine protected areas around remote islands, such as Rapa Nui. In addition, the mitogenomes presented here have potential implications for future biomedical research because antipatharians have been used for medicinal purposes in many cultures (Wagner et al. 2012).

**Figure 1.**
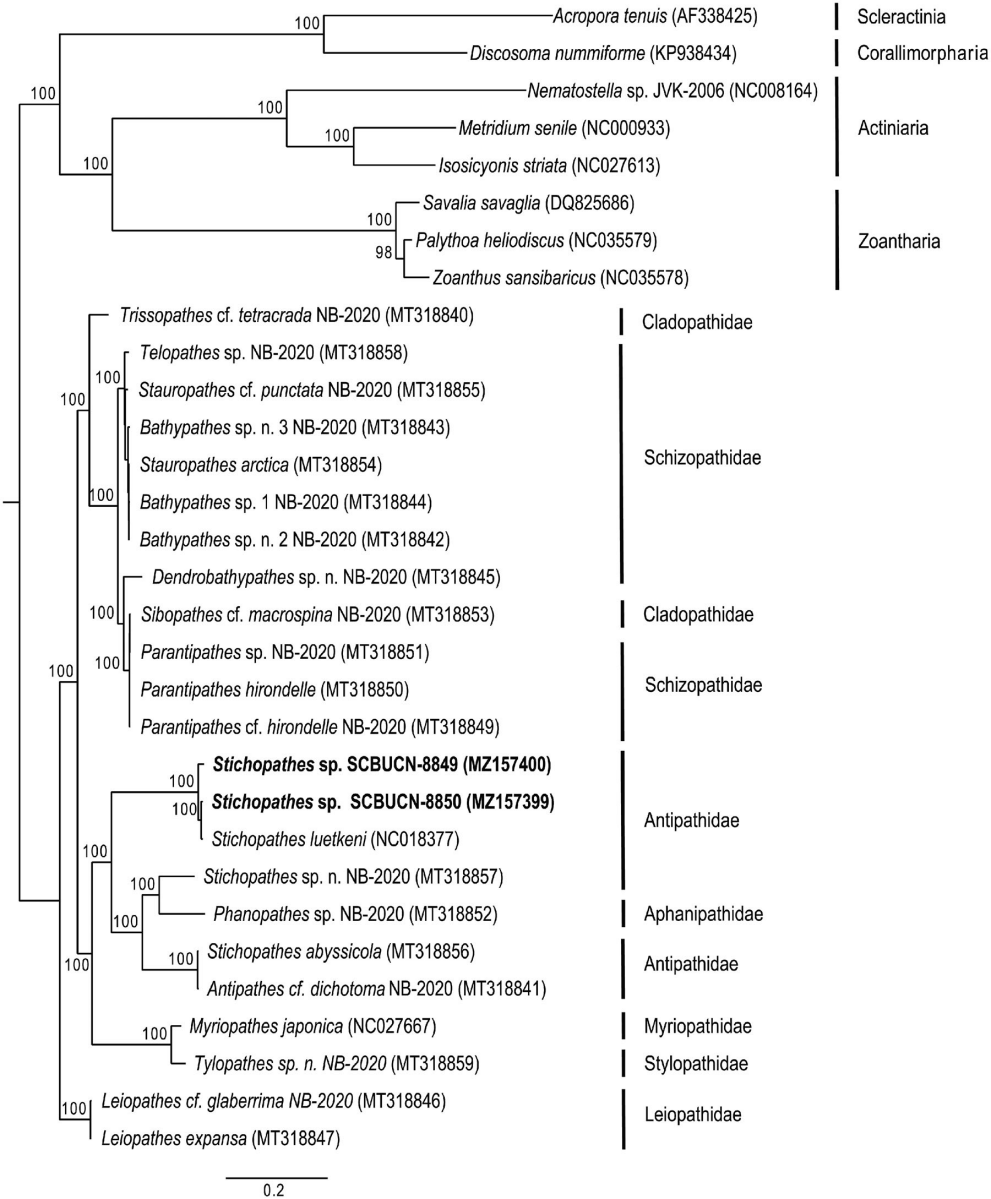
Maximum-likelihood, phylogenetic reconstruction based on the complete mitochondrial proteome of two *Stichopathes* species (in bold) collected from Rapa Nui (Easter Island) and 21 representatives of Antipatharia and eight representatives from other Hexacorallia subclasses as outgroups: Scleractinia, Corallimorpharia, Actiniaria, and Zoantharia. Species names and GenBank accession numbers are included in parentheses at the tips. All nodes had bootstraps ≥ 76, but values were excluded in large, densely branched clades. See Supplemental data for details.

## Supplementary Material

Supplemental Material

## Data Availability

The mitochondrial genome sequence data that support the findings of this study are openly available in GenBank of NCBI at https://www.ncbi.nlm.nih.gov under the accession no. MZ157399-MZ157400. The associated voucher repository (Sala de Colecciones, UCN, Javier Sellanes, sellanes@ucn.cl) numbers are SCBUCN-8850 and SCBUCN-8849 respectively.
