# Supplementary material for: Complete mitochondrial genomes of two species of Stichopathes Brook, 1889 (Hexacorallia: Antipatharia: Antipathidae) from Rapa Nui (Easter Island)
Source: Mitochondrial DNA B Resour. 2021 Oct 15;6(11):3226–8. doi: 10.1080/23802359.2021.1990150 (PMC8525922; doi:10.1080/23802359.2021.1990150)
Supplement: Supplemental Material [file TMDN_A_1990150_SM2228.docx]

**Supplemental files for the publication**

**Complete mitochondrial genomes of two species of *Stichopathes* Brook, 1889 (Hexacorallia: Antipatharia: Antipathidae) from Rapa Nui (Easter Island)**

**Cynthia M. Asorey^1^, Javier Sellanes^1^, Daniel Wagner^2^, Erin E. Easton^3^**

**^1^Millennium Nucleus for Ecology and Sustainable Management of Oceanic Islands**

**(ESMOI), Departamento de Biología Marina, Facultad de Ciencias del Mar and Sala de Colecciones Biológicas, Universidad Católica del Norte, Larrondo 1281, Coquimbo, Chile.**

**^2^Conservation International, Center for Oceans, Arlington, VA, United States**

**^3^School of Earth, Environmental and Marine Sciences, University of Texas Rio Grande**

**Valley, 33363 Marine Lab Dr., South Padre Island, TX 78597, United States.**

**Submitted to Mitochondrial DNA Part B: 14 July 2021**

**Specimens deposited in the Sala de colecciones Biológicas at the Universidad Católica del Norte (SCBUCN):**

**SCBUCN-8849, *Stichopathes* sp. (yellow morphotype)**

**SCBUCN-8850, *Stichopathes* sp. (red morphotype)**

**Completes mitogenome sequence deposited to GenBank:**

**MZ157399,** ***Stichopathes* sp. SCBUCN-8849 (yellow morphotype)**

**MZ157400, *Stichopathes* sp. SCBUCN-8850 (red morphotype)**

**Correspondence details: UTRGV-SEEMS, 33363 Marine Lab Dr., South Padre Island, TX 78597;** [**erin.easton@utrgv.edu**](mailto:erin.easton@utrgv.edu)

**Methods**

The two specimens of whip black corals were collected by chance, tangled in the propellers of a Commander MK2 remotely operated vehicle (Mariscope Meerestechnik, Kiel, Germany). The specimens were stored in 95% ethanol and deposited in the Sala de Colecciones, Universidad Católica del Norte, Chile (SCBUCN). The yellow morphotype (SCBUCN 8849) was collected between 120 and 180 m (-27.100º, -109.431º) on 8 April 2017 and the red morphotype (SCBUCN 8850) was collected at 180 m (-27.101º -109.426º) on 9 April 2017. Polyp arrangement on both specimens was in a single row on only one side of the corallum, a diagnostic morphological character currently assigned to the genus *Stichopathes* (Bo & Opresko 2015). Genomic DNA was isolated from a single polyp with GeneJET Genomic DNA Purification Kit (ThermoFisher Scientific, Waltham, MA) per manufacture’s protocol. Isolated DNA was submitted to Biopolymers Facility at Harvard Medical School for library preparation and next-generation sequencing (NextSeq 500). Trimmed reads (Trimmomatic-0.32, Bolger et al. 2014) were assembled *de novo* by SPAdes (Bankevich et al. 2012) on the University of New Hampshire Bioinformatics Core facility ron server, which is supported by the New Hampshire-INBRE Program through an Institutional Development Award (IDeA), P20GM103506, from the National Institute of General Medical Sciences of the NIH(see Trimmomatic and SPAdes scripts below for settings). To identify the mitochondrial genome, we conducted a blastn search of the resulting SPAdes contigs against all reference mitochondrial genomes in GenBank downloaded to the ron server. The identified contig was then imported into Geneious Prime 2021.1.1 (<https://www.geneioius.com>) and circularized. We used the find repeats option in Geneious to identify any repeats >15 bp. Consecutive repeats at the junction of the ends of the SPAdes contig were reviewed manually and one repeat, presumed to be an artefact of assembly, was deleted. To confirm this assumption and for quality assurance and control, reads were mapped to this sequence in Geneious as follows. Reads were paired with default settings for Illumina paired ends as implemented in Geneious except Expected Distance = 150. Paired reads were trimmed with BBDuk v. 38.84as implemented in Geneious. BBDuk default settings were used except the following options were selected (see below for commands): Trim adapters (Ilumina Nextera adapters – 71 sequences) and Trim Low Quality. BBDuk-trimmed reads were mapped to the edited SPAdes contig in Geneious to generate a consensus sequence with the following settings: Mapper = Geneious, Sensitivity = Medium-Low Sensitivity, Fine Tuning = Iterate up to 5 times, Results = all boxes checked with default Assembly Name (Save Consensus options = 95%, Highest Quality, and Assign Quality Total), Trim Before Mapping = Do Not Trim, Map multiple best matches = Randomly, Trim paired read overhangs = checked, Allow Gaps = checked (Maximum Per Read = 10%, Maximum Gap Size = 15), Word length = 18, Index word length = 13, Ignore words repeated more than 12 times = checked, Maximum mismatches per read = 20%, Maximum Ambiguity = 4, and Accurately map reads with errors to repeat regions = checked.

Genes were annotated by mapping *Stichopathes luetkeni* (Antipathidae; GenBank accession NC018377) annotations and manually adjusting them in Geneious. For 12S and 16S rRNA genes, a consistent start and end position was not observed among members of the Antipatharian, so we retained the annotations based on *S. luetkeni* annotations. When alternative start codons were identified for protein-coding genes, we selected the start codon that agreed with *S. luetkeni* because it is a reference mitochondrial genome and it was the most genetically similar to our *Stichopathes* spp*.* However, several alternative start codons were identified that started with ATG and were more commonly identified as start codons in other antipatharians (see Alternative Start codons section below for more information). The mitogenome protein-coding genes were concatenated and then aligned with default MUSCLE (Edgar 2004) parameters in Geneious to all antipatharian representative species for which the complete mitochondrial genome was available in GenBank: *Antipathes cf. dichotoma* NB-2020 (MT318841), *Bathypathes* sp. 1 NB-2020 (MT318844), *Bathypathes* sp. n. 2 NB-2020 (MT318842), *Bathypathes* sp. n. 3 NB-2020 (MT318843), *Dendrobathypathes* sp. n. NB-2020 (MT318845), *Leiopathes* cf. *glaberrima* NB-2020 (MT318846), *Leiopathes expansa* (MT318847), *Myriopathes japonica* (NC027667), *Parantipathes* cf. *hirondelle* NB-2020 (MT318849), *Parantipathes hirondelle* (MT318850), *Parantipathes* sp. NB-2020 (MT318851), *Phanopathes* sp. NB-2020 (MT318852), *Sibopathes* cf. *macrospina* NB-2020 (MT318853), *Stauropathes arctica* (MT318854), *Stauropathes* cf. *punctata* NB-2020 (MT318855), *Stichopathes abyssicola* (MT318856), *Stichopathes luetkeni* (NC018377), *Stichopathes* sp. n. NB-2020 (MT318857), *Telopathes* sp. NB-2020 (MT318858), *Trissopathes* cf. *tetracrada* NB-2020 (MT318840), and *Tylopathes* sp. n. NB-2020 (MT318859). Following Barret et al (2020), we selected eight representative from other Hexacorallia subclasses as outgroups: Scleractinia - *Acropora tenuis* (AF338425); Coralliomorpharia - *Discosoma nummiforme* (KP938434); Actiniaria - *Isosicyonis striata* (NC027613), *Metridium senile* (NC000933), and *Nematostella* sp. JVK-2006 (NC008164); and Zoantharia - *Palythoa heliodiscus* (NC035579), *Savalia savaglia* (DQ825686), and *Zoanthus sansibaricus* (NC035578). To select the best model of nucleotide evolution, we used PartitionFinder 2.1.1 (Lanfear et al. 2017) with the following settings defined in Scripts and commands (below) and data blocks defined to the common extent of each protein-coding gene among all taxa in the alignment split into the three codon positions (see Scripts and commands below). A maximum-likelihood, phylogenetic tree was constructed in RAxML 8.2.11 (Stamatakis 2014): 1000 bootstrap replicates (rapid bootstrapping with search for best-scoring ML tree), nucleotide model = GTR GAMMA I, with partitions identified in PartitionFinder (See Scripts and commands below).

The ITS1-based reconstruction is available from the corresponding author or in Tapia-Guerra et al. (in review). Methods (in brief) for the ITS1-based reconstruction follow. Sequences of 70 antipatharians and the scleractinians *Porites lutea* were aligned with default MUSCLE 3.8.425 (Edgar, 2004) parameters in Geneious Prime. The alignment was trimmed to the extent of the shortest sequences, resulting in a 343-bp alignment. ModelTest-NG 0.1.6 (Darriba et al., 2019) was used to select the substitution models applicable to PhyML phylogenetic reconstructions with the following settings: Data type=DNA, Templates=PhyML, models=11, Rate variation=uniform/gamma/pinv/+I+G, and Frequencies=Equal frequencies/ML frequencies. A maximum-likelihood, phylogenetic tree was constructed with PhyML 3.3.20180621 (Guindon et al., 2010) plugin of Geneious Prime with the following settings: substitution model=TPM3+G4, bootstrap=1000, and optimize=topology/length/rate.

**Scripts and commands**

Trimmomatic script:

#1/bin/bash

for dir in $@

do

cat $dir/*R1*.fastq > $dir/combined-1.fastq

cat $dir/*R2*.fastq > $dir/combined-2.fastq

FORWARD=$dir/combined-1.fastq;

REVERSE=$dir/combined-2.fastq;

FORWARD=$(echo $FORWARD);

REVERSE=$(echo $REVERSE);

nohup trimmomatic PE -threads 8 $FORWARD $REVERSE $dir/paired-1.fastq.gz $dir/unpaired-1.fastq.gz $dir/paired-2.fastq.gz $dir/unpaired-2.fastq.gz ILLUMINACLIP:/opt/Trimmomatic-0.32/adapters/NexteraPE-PE.fa:2:30:10 LEADING:3 TRAILING:3 SLIDINGWINDOW:4:15 MINLEN:3$

done

Spades script:

for dir in $@

do

cd $dir

FORWARD=paired-1.fastq.gz

REVERSE=paired-2.fastq.gz

UNPAIRED1=unpaired-1.fastq.gz

UNPAIRED2=unpaired-2.fastq.gz

nohup spades.py --pe1-1 $FORWARD --pe1-2 $REVERSE --pe1-s $UNPAIRED1 --pe1-s $UNPAIRED2 -t 16 -o spades_assembly &

cd ../

done

BBDuk commands:

[ktrim=r, k=27, hdist=1, edist=0, ref=nextera.fa.gz, qtrim=rl, trimq=6, minlength=10, ordered=t, qin=33, in=input1.fastq, in2=input2.fastq, out=output1.fastq, out2=output2.fastq]

PartitionFinder settings and RaxML commands per PartitionFinder:

branchlengths: linked

models: JC, K80, TRNEF, K81, TVMEF, TIMEF, SYM, F81, HKY, TRN, K81UF, TVM, TIM, GTR, JC+G, K80+G, TRNEF+G, K81+G, TVMEF+G, TIMEF+G, SYM+G, F81+G, HKY+G, TRN+G, K81UF+G, TVM+G, TIM+G, GTR+G, JC+I, K80+I, TRNEF+I, K81+I, TVMEF+I, TIMEF+I, SYM+I, F81+I, HKY+I, TRN+I, K81UF+I, TVM+I, TIM+I, GTR+I, JC+I+G, K80+I+G, TRNEF+I+G, K81+I+G, TVMEF+I+G, TIMEF+I+G, SYM+I+G, F81+I+G, HKY+I+G, TRN+I+G, K81UF+I+G, TVM+I+G, TIM+I+G, GTR+I+G

model_selection: aicc

search: greedy

(Lanfear et al. 2012)

Best partitioning scheme

Scheme Name: step_26

Scheme lnL: -83273.13177490234

Scheme AICc: 166958.829067

Number of params: 203

Number of sites: 12819

Number of subsets: 16

Scheme Description in PartitionFinder format

Scheme_step_26 = (NAD1_1stpos, NAD4L_1stpos, NAD2_1stpos, Atp6_1stpos, NAD6_1stpos, COII_1stpos) (NAD6_2ndpos, Atp6_2ndpos) (NAD4L_3rdpos, ATP8_3rdpos, COII_3rdpos, Atp6_3rdpos, NAD6_3rdpos, NAD4_3rdpos) (ATP8_1stpos, NAD3_1stpos, ATP8_2ndpos) (COI_1stpos) (COI_2ndpos) (COI_3rdpos, CYTB_3rdpos, COIII_3rdpos) (COII_2ndpos, NAD4L_2ndpos) (NAD5_1stpos, NAD4_1stpos, CYTB_1stpos, COIII_1stpos) (NAD5_2ndpos, NAD2_2ndpos, CYTB_2ndpos, COIII_2ndpos, NAD4_2ndpos) (NAD3_2ndpos, NAD1_2ndpos) (NAD3_3rdpos, NAD5_3rdpos, NAD1_3rdpos) (NAD2_3rdpos) (HEG_1stpos) (HEG_2ndpos) (HEG_3rdpos);

RaxML-style partition commands

DNA, Subset1 = 5032-6036\3, 7540-7839\3, 6037-7182\3, 1-639\3, 11182-11775\3, 2380-3120\3

DNA, Subset2 = 11183-11775\3, 2-639\3

DNA, Subset3 = 7542-7839\3, 642-858\3, 2382-3120\3, 3-639\3, 11184-11775\3, 7842-9354\3

DNA, Subset4 = 640-858\3, 7183-7539\3, 641-858\3

DNA, Subset5 = 859-2379\3

DNA, Subset6 = 860-2379\3

DNA, Subset7 = 861-2379\3, 3912-5031\3, 3123-3909\3

DNA, Subset8 = 2381-3120\3, 7541-7839\3

DNA, Subset9 = 9355-11181\3, 7840-9354\3, 3910-5031\3, 3121-3909\3

DNA, Subset10 = 9356-11181\3, 6038-7182\3, 3911-5031\3, 3122-3909\3, 7841-9354\3

DNA, Subset11 = 7184-7539\3, 5033-6036\3

DNA, Subset12 = 7185-7539\3, 9357-11181\3, 5034-6036\3

DNA, Subset13 = 6039-7182\3

DNA, Subset14 = 11776-12819\3

DNA, Subset15 = 11777-12819\3

DNA, Subset16 = 11778-12819\3

**Alternative start codons**

We found alternative start codons (ATG) for the following genes:

1. **ND4**

Chosen start: position 4354 in red morphotype, *Stichopathes* sp. SCBUCN-8850, and 4433 in yellow morphotype, *Stichopathes* sp. SCBUCN-8849.

Alternative start: 27 bp downstream; position 4381 in red morphotype, *Stichopathes* sp. SCBUCN-8850, and 4460 in yellow morphotype, *Stichopathes* sp. SCBUCN-8849.

Except for *Stichopathes luetkeni*, none of the other antipatharian species start at the chosen position because a stop codon exists between the chosen and alternative start, which was the start identified in the other species.

1. **ND1**

Chosen start: position 9099 in red morphotype, *Stichopathes* sp. SCBUCN-8850, and 9019 in yellow morphotype, *Stichopathes* sp. SCBUCN-8849.

Alternative start 1: 9 bp downstream at 9108 in red morphotype, *Stichopathes* sp. SCBUCN-8850, and 9028 in yellow morphotype, *Stichopathes* sp. SCBUCN-8849.

Alternative start 2: 66 bp downstream at 9165 in red morphotype, *Stichopathes* sp. SCBUCN-8850, and 9085 in yellow morphotype, *Stichopathes* sp. SCBUCN-8849.

As with ND4, the other Antipatharia species, except *S. luetkeni*, have stop codons between the chosen start and second alternative start, so the downstream alternative position is the one conserved among species.

1. **ATP6**

Chosen start: position 7258 in red morphotype, *Stichopathes* sp. SCBUCN-8850, and 7179 in yellow morphotype, *Stichopathes* sp. SCBUCN-8849.

Alternative start: 66 bp upstream at 7192 in red morphotype, *Stichopathes* sp. SCBUCN-8850, and 7113 in yellow morphotype, *Stichopathes* sp. SCBUCN-8849.

This alternative start is the start identified in other antipatharian species, but not in *S. luetkeni*, which has a stop codon near the alternative start. Unlike *S. luetkeni,* our *Stichopathes* sp. do not have this stop codon.

**
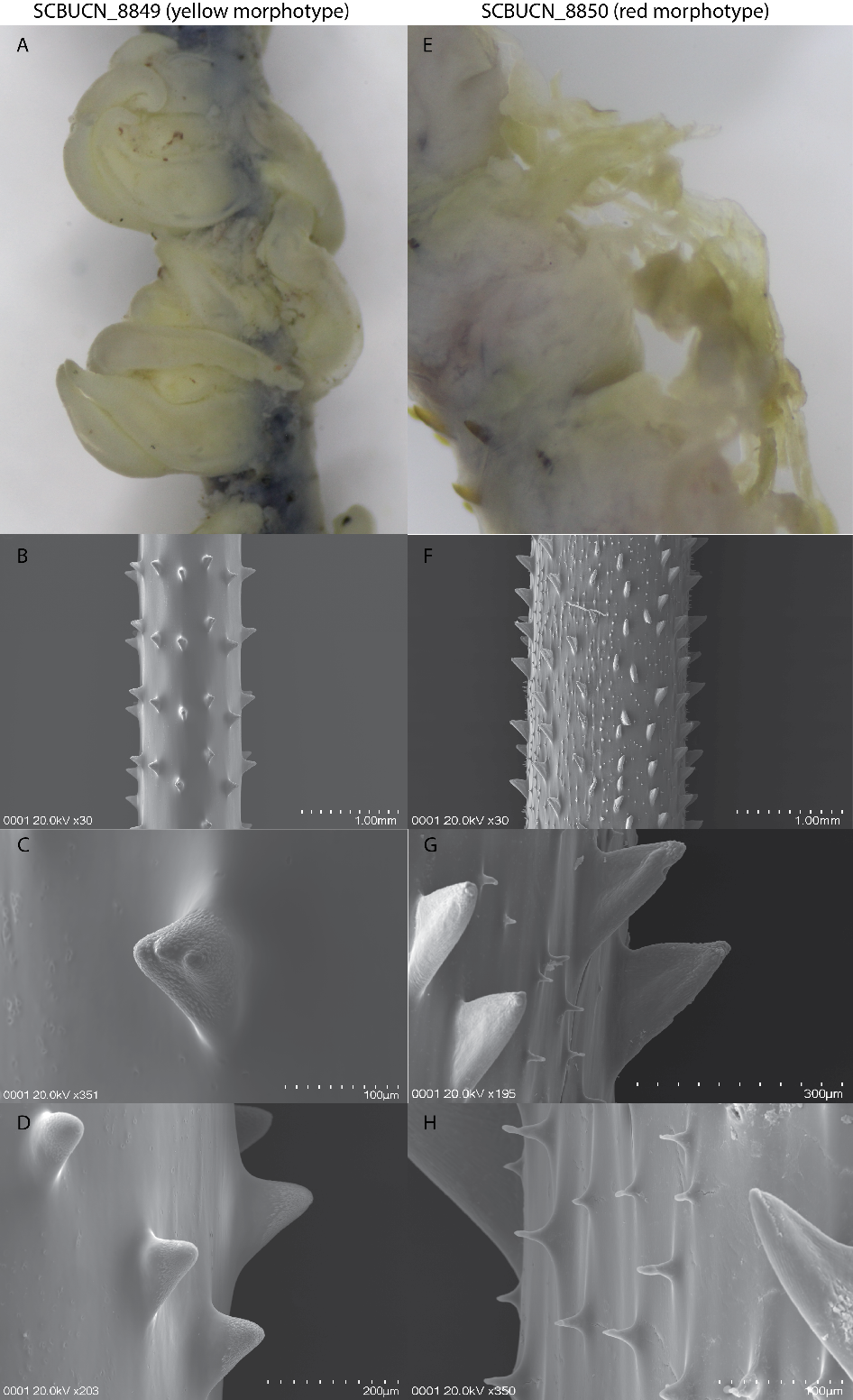
**

**Figure S1.** Morphological characteristics of polyps, corallum, and spines in the yellow  morphotype (*Stichopathes* sp. SCBUCN- 8849) (**A-D**) and red morphotype (*Stichopathes* sp. SCBUCN- 8850) (**E-H**). **A, E** polyps on basal branch under light microscopy; **E** polyps in poor condition; **D-C, F-H** close-up view of the basal region of the stem, showing the presence of secondary spines on the stem of red morphotype (*Stichopathes* sp. SCBUCN- 8850); **C-D, G-H** close-up view of polypar spines on basal region of the stem (also see Figure 3 in Tapia-Guerra et al. *in review* or contact the corresponding author for these data and supporting morphological data associated with Tapia-Guerra et al.).

**Video S1.** Video clips of each whip coral morphotype as well as a video clip of the sea star (cf. *Ceramaster australis*) feeding on a fallen coral of yellow morphotype. Video taken with an ROV on Rapa Nui and Pukao Seamount at ~120-270 m (T2- 2017). Video credits: Matthias Gorny (OCEANA)

<https://www.youtube.com/watch?v=fOIx7tGGvKI>

**Acknowledgements**

We thank Poky Tane Haoa, Ricardo Hito and Enrique Hey

from the Rapa Nui community for their collaboration during field work. Special thanks to Sergio Rapu and the Rapa Nui Heritage Foundation for providing land and facilities for our on-island laboratory. The present study was funded in part by ANID – Millennium Science Initiative Program – NC120030, OCEANA (providing also the ROV), and FONDECYT 1181153 and 1180694 grants to JS. Additional support was provided through the Coral Reefs of the High Seas Coalition by Conservation International, the Paul M. Angell Foundation, Alan Eustace, and Tom and Currie Barron

**Permissions**

Sample collection was performed under permissions Res. Ext N°41/2016 and N°3314/2017 from SUBPESCA (National Fishing Authority of Chile) to Universidad Católica del Norte. This research was also presented to the local “Consejo del Mar de Rapa Nui” (Council of the Sea of Rapa Nui), which allowed sampling around the island.

**Literature cited:**

Bankevich A, Nurk S, Antipov D, Gurevich AA, Dvorkin M, Kulikov AS, Lesin VM, Nikolenko SI, Pham S, Prjibelski AD. 2012. SPAdes: a new genome assembly algorithm and its applications to single-cell sequencing. J Comput Biol. 19(5):455-477.

Barrett, N.J., Hogan, R.I., Allcock, A.L., Molodtsova, T., Hopkins, K., Wheeler, A.J., Yesson, C. 2020. Phylogenetics and Mitogenome Organisation in Black Corals (Anthozoa: Hexacorallia: Antipatharia): An Order-Wide Survey Inferred From Complete Mitochondrial Genomes. *Frontiers in Marine Science*, **7**.

Bo M & Opresko DM 2015. Redescription of Stichopathes pourtalesi Brook, 1889 (Cnidaria: Anthozoa: Antipatharia: Antipathidae). Breviora. 540: 1–18.

Bolger AM, Lohse M, Usadel B. 2014. Trimmomatic: a flexible trimmer for Illumina sequence data. Bioinformatics. 30(15):2114-2120.

Darriba, D., Posada, D., Kozlov, A. M., Stamatakis, A., Morel, B., and Flouri, T. (2019). ModelTest-NG: A new and scalable tool for the selection of DNA and protein evolutionary models. bioRxiv, 1–6. doi:10.1101/612903.

Edgar RC. 2004. MUSCLE: multiple sequence alignment with high accuracy and high throughput. Nucleic Acids Res. 32(5):1792-1797.

Guindon, S., Dufayard, J. F., Lefort, V., Anisimova, M., Hordijk, W., and Gascuel, O. (2010). New algorithms and methods to estimate maximum-likelihood phylogenies: Assessing the performance of PhyML 3.0. Syst. Biol. 59, 307–321. doi:10.1093/sysbio/syq010.

Lanfear R, Calcott B, Ho SY, Guindon S. 2012. PartitionFinder: combined selection of partitioning schemes and substitution models for phylogenetic analyses. Molecular biology and evolution. 29(6):1695-1701.

Lanfear R., Frandsen PB, Wright AM, Senfeld T, Calcott B. 2017 PartitionFinder 2: New Methods for Selecting Partitioned Models of Evolution for Molecular and Morphological Phylogenetic Analyses.Molecular Biology and Evolution. 34(3): 772–773

Stamatakis A. 2014. RAxML version 8: a tool for phylogenetic analysis and post-analysis of large phylogenies. Bioinformatics. 30(9):1312-1313.

Tapia-Guerra JM, Asorey CM, Easton EE, Wagner D, Gorny M, Sellabes J. In review. First ecological characterization of whip black coral assemblages (Hexacorallia: Antipatharia) in the Easter Island Ecoregion, southeastern Pacific. Frontiers in Marine Science.
